# Social and Nonsocial Relational Reasoning in Adolescence and Adulthood

**DOI:** 10.1162/jocn_a_01153

**Published:** 2017-05-31

**Authors:** Lucía Magis-Weinberg, Sarah-Jayne Blakemore, Iroise Dumontheil

**Affiliations:** 1 University College London; 2 University of London

## Abstract

Reasoning during social interactions requires the individual manipulation of mental representations of one’s own traits and those of other people as well as their joint consideration (relational integration). Research using nonsocial paradigms has linked relational integration to activity in the rostrolateral PFC. Here, we investigated whether social reasoning is supported by the same general system or whether it additionally relies on regions of the social brain network, such as the medial PFC. We further assessed the development of social reasoning. In the social task, participants evaluated themselves or a friend, or compared themselves with their friend, on a series of traits. In the nonsocial task, participants evaluated their hometown or another town or compared the two. In a behavioral study involving 325 participants (11–39 years old), we found that integrating relations, compared with performing single relational judgments, improves during adolescence, both for social and nonsocial information. Thirty-nine female participants (10–31 years old) took part in a neuroimaging study using a similar task. Activation of the relational integration network, including the rostrolateral PFC, was observed in the comparison condition of both the social and nonsocial tasks, whereas the medial PFC showed greater activation when participants processed social as opposed to nonsocial information across conditions. Developmentally, the right anterior insula showed greater activity in adolescents compared with adults during the comparison of nonsocial versus social information. This study shows parallel recruitment of the social brain and the relational reasoning network during the relational integration of social information in adolescence and adulthood.

## Introduction

Is London more expensive than Cambridge? Answering this question entails at least two levels of relational reasoning. At the first level, one needs to judge the prices in each city independently (evaluation of single relations, e.g., how much do houses in London cost?). At the second level, one needs to simultaneously consider mental representations of both cities and to integrate the single judgments into a higher-order comparison (relational integration, in this case, comparing the house prices in London and Cambridge). Relational integration has typically been studied in nonsocial contexts, in particular, using the Raven’s Progressive Matrices (Raven, 1998). However, relational integration also occurs in the social domain, for example, when comparing people on personality traits (e.g., are you more patient than your friend?). The neural processes supporting this kind of social reasoning and the way it develops are not well understood.

Previous fMRI research has identified the lateral PFC and lateral parietal cortex as involved in relational integration (Dumontheil, 2014; Bunge, Helskog, & Wendelken, 2009; Wendelken, Nakhabenko, Donohue, Carter, & Bunge, 2008; Smith, Keramatian, & Christoff, 2007), whereas medial prefrontal cortex (MPFC) has been associated with the processing and manipulation of social information (Van Overwalle, 2009; Gilbert et al., 2006; Wood & Grafman, 2003). The current study aimed to bring together these separate strands of research to investigate domain-general and social domain-specific processes that support the relational integration of social information. Both relational reasoning and social cognition and their underlying neural substrates undergo significant reorganization during adolescence (Kilford, Garrett, & Blakemore, 2016; Dumontheil, 2014). Therefore, a second aim of the current study was to compare social reasoning in adolescents and adults. We employed a paradigm that allows the investigation and comparison of relational integration of both social and nonsocial information (Raposo, Vicens, Clithero, Dobbins, & Huettel, 2011). In a large behavioral study, we investigated the development of relational integration of social and nonsocial information from late childhood until adulthood. In a follow-up fMRI study, we studied the neural correlates of these cognitive processes in adolescence and adulthood.

### Neural Bases of Relational Integration and Social Cognition

Relational reasoning research suggests a central role of rostrolateral prefrontal cortex (RLPFC), which corresponds to the lateral aspect of the anterior, or rostral, prefrontal cortex (Brodmann’s areas [BAs] 10/46 and 10/47), in relational integration compared with processing single relations. Imaging studies using the Raven’s Progressive Matrices in adults have shown RLPFC involvement in the joint manipulation of visuospatial patterns (Kroger et al., 2002; Christoff et al., 2001) as well as in the integration of relations in analogical reasoning tasks (Wendelken et al., 2008; Bunge, Wendelken, Badre, & Wagner, 2005) and in the integration of multiple relations to reach a logical conclusion (Wendelken & Bunge, 2009). A study comparing visuospatial and semantic variants of a relational matching task found considerable activation overlap within the left RLPFC, suggesting a domain-general role for RLPFC in relational integration (Wendelken, Chung, & Bunge, 2012).

Social cognitive research suggests a role of the MPFC, which corresponds to BA 8/BA 9/BA 10, in the processing of social information (see Van Overwalle, 2009, for a meta-analysis). Studies with adults have shown that this region is involved in considering one’s thoughts and feelings (Rameson, Satpute, & Lieberman, 2010; Gusnard, 2005; Zysset, Huber, Samson, Ferstl, & von Cramon, 2003) and in perspective taking (PT; David et al., 2008; D’Argembeau et al., 2007; Aichhorn, Perner, Kronbichler, Staffen, & Ladurner, 2006; David et al., 2006; Ruby & Decety, 2001, 2004; Vogeley et al., 2004). The MPFC is also recruited during tasks that require mentalizing, that is, the consideration of other people’s mental states (Amodio & Frith, 2006; Decety & Sommerville, 2003; Frith & Frith, 2003).

### Development during Adolescence

Both relational integration and social cognition show protracted development in terms of improved performance and associated brain activity between adolescence and adulthood (Blakemore, 2012; Crone & Dahl, 2012; Dumontheil & Blakemore, 2012; Dumontheil, Hillebrandt, Apperly, & Blakemore, 2012; Crone et al., 2009; Dumontheil, Burgess, & Blakemore, 2008). The RLPFC undergoes structural and functional development with age, with evidence that its activity during relational integration tasks becomes increasingly specialized during childhood and adolescence (Dumontheil, 2014; Wendelken, O’Hare, Whitaker, Ferrer, & Bunge, 2011; Dumontheil, Houlton, Christoff, & Blakemore, 2010; Crone et al., 2009; Ferrer, O’Hare, & Bunge, 2009; Dumontheil et al., 2008). In addition, a complex pattern of developmental changes in functional connectivity related to reasoning ability has been identified, including changes in connectivity between the RLPFC and the parietal cortex (Wendelken, Ferrer, Whitaker, & Bunge, 2016; Bazargani, Hillebrandt, Christoff, & Dumontheil, 2014). Bazargani et al. (2014) observed a decrease in short-range (fronto-insular) connectivity with stable long-range connectivity (frontoparietal) and an increase of modulatory connections with age. Wendelken et al. (2016) found a pattern of developmental changes suggestive of increasing communication between prefrontal regions and specific targets.

Key regions of the social brain, including the MPFC, undergo structural and functional changes during adolescence. Cortical thickness and gray matter volume in the MPFC decrease between late childhood and the early 20s (Mills, Lalonde, Clasen, Giedd, & Blakemore, 2014). In parallel, several fMRI studies have shown that MPFC activity during mentalizing tasks decreases between early adolescence and adulthood (Blakemore, 2008, 2012). In a previous study investigating the development of the neural correlates of mentalizing, participants were required either to take someone else’s perspective or to use symbolic cues to select an appropriate action in a communicative context. We found that adolescents showed hypoactivation of domain-general cognitive control regions in the parietal cortex and PFC and hyperactivation of parts of the social brain network (Dumontheil et al., 2012). This study thus demonstrated the engagement of cognitive control and social brain regions within a single paradigm and that the engagement of these regions changes as a function of age.

Relational integration within the social domain has been investigated in adults using a task that combined both mentalizing and relational integration (Raposo et al., 2011). Participants judged how pleasant they found a certain word, how pleasant a friend would find the word, and how their rating of pleasantness would compare with that of their friend. Behaviorally, RTs were higher when participants were comparing themselves with their friend relative to the two single-relation conditions. MPFC activation was higher during the friend judgment compared with the self-judgment, whereas RLPFC activation was higher when contrasting the relational integration comparison and self-judgment conditions. The study did not include a nonsocial relational reasoning condition, preventing the conclusion that the activation patterns are specific to relational integration of social information per se or reflective of relational integration more generally.

### This Study

Here, we adapted the paradigm designed by Raposo et al. (2011) to investigate behavioral development of social reasoning (Study 1) and its neural development between adolescence and adulthood (Study 2). We compared first-order judgments (1-REL) of traits associated with oneself or with another individual (e.g., How patient are you? [Self condition]; How patient is your friend? [Other condition]) with second-order judgments (2-REL) about how these judgments related to each other (How much more patient are you than your friend? [Comparison condition]). Our paradigm also included a control nonsocial task, in which participants were asked to rate characteristics of towns. Our aim was to assess (1) how performance on a task requiring relational integration of social or nonsocial traits develops between late childhood and adulthood, (2) how neural activity underlying these processes develops between early adolescence and adulthood, and (3) whether there is domain-specific activation for the relational integration of social versus nonsocial information.

In terms of behavior, we predicted improvements in relational integration with age, both in terms of RT and the consistency of participants’ responses between 1-REL and 2-REL judgments. In terms of BOLD signal, we expected domain-general activations associated with relational integration in the RLPFC, dorsolateral PFC, and parietal cortex. We also predicted that there would be additional domain-specific activations in parts of the social brain network associated with the people task, specifically regions involved in processing social information and mentalizing (Meyer, Taylor, & Lieberman, 2015; Dumontheil & Blakemore, 2012; Meyer, Spunt, Berkman, Taylor, & Lieberman, 2012; Raposo et al., 2011). Finally, we predicted that the RLPFC would show increased specificity of activation for 2-REL versus 1-REL judgments in adults compared with adolescents (Dumontheil, 2014) and that the MPFC would show greater activation in adolescents than adults in the social versus nonsocial task (Blakemore & Robbins, 2012; Blakemore, 2008).

## Study 1: Behavioral Study

### Participants

The data analyzed here are part of a larger project in male and female children, adolescents, and adults who performed a set of six tasks and provided saliva samples for genetic analyses (Kilford, Dumontheil, Wood, & Blakemore, 2015; Dumontheil et al., 2014). The present analysis focused on the social and nonsocial comparison task and the Wechsler Abbreviated Scale of Intelligence (WASI; Wechsler, 1999) assessment. The data presented here are from 325 participants aged between 11 and 39 years (*n* = 160 adults, *n* = 165 children and adolescents). From an original sample of 399 participants, one was excluded because of a diagnosis of Turner syndrome, one was excluded because of a diagnosis of Asperger syndrome, four were excluded because of a task programming error, two were excluded because they interrupted the task early, one participant did not have time to complete this task, and data were lost from 15 participants. In addition, as only four male participants were younger than 11 years (vs. 26 female participants), all participants younger than 11 years were excluded from further analyses. Children and adolescents were recruited from schools in and around London and were tested in their school, and adults were recruited from the University College London (UCL) Psychology Department volunteer database and word of mouth and tested in the laboratory. Written informed consent was obtained from participants or from the parent/guardian of participants under 18 years old. Adult participants were remunerated for their time. The study was approved by the UCL ethics committee.

Child and adolescent participants were divided according to age into three groups spanning 2 or 3 years, and there were four adult groups (Table 1). Verbal ability was measured using the vocabulary subtest of the WASI (Wechsler, 1999). A two-way (Age group, Sex) ANOVA indicated that there was a significant difference in verbal IQ between age groups (*F*(1, 307) = 2.91, *p* = .009). Paired post hoc comparisons demonstrated that the age groups of 11–12 and 13–14 years had lower mean verbal IQ than the age group of 20–22 years (*p*s < .05) and that the age group of 26–28 years had lower mean verbal IQ than all other age groups (*p*s < .05; Table 1). Only the difference between the age groups of 20–22 and 26–28 years survived Bonferroni correction (*p* = .001). There was no main effect of Sex on IQ (*F*(1, 307) = 2.34, *p* = .127), but there was a significant Sex × Age group interaction (*F*(6, 307) = 3.08, *p* = .006). Post hoc comparisons of male and female participants in each age group indicated that 11- to 12-year-old male participants had lower mean verbal IQ (109.1, *SD* = 12.2) than female participants (118.1, *SD* = 10.5, *p* = .002), whereas 23- to 25-year-old men had higher mean verbal IQ (118.6, *SD* = 11.1) than women (109.7, *SD* = 14.5, *p* = .023).

### Design and Stimulus Material

The task had two within-participant factors (Task: people or town; Condition: self, other, or comparison) and one between-participant factor (Age group: seven levels), resulting in a 2 × 3 × 7 mixed factorial design. The task was computer based and adapted from the fMRI study by Raposo et al. (2011). The task was administered as part of a single individual testing session of approximately 45–50 min. It was the third task administered in the task set, and the WASI was administered as the sixth (and last) task.

The experimenter started by asking participants to think of someone whom they knew quite well but who was quite different from them and to give his or her name. If participants did not respond, the experimenter suggested that they consider a close friend or a sibling who was quite different from them. Second, participants were asked to name the town where they lived (typically, London) and then pick a town that they knew quite well but that was quite different from London. Again, if participants did not respond, the experimenter suggested that they consider a town where they go on a holiday or where their grandparents live. Instructions were then presented on the screen and read aloud to the participants, explaining the different types of judgment they would make during the task and the rating scale. Participants used the index, middle, and ring fingers of both hands to respond. The task was programmed in Cogent (www.vislab.ucl.ac.uk/cogent_graphics.php) running in MATLAB (The MathWorks, Inc., Natick, MA) on a Dell 12-in. laptop or similar.

**Figure 1:**
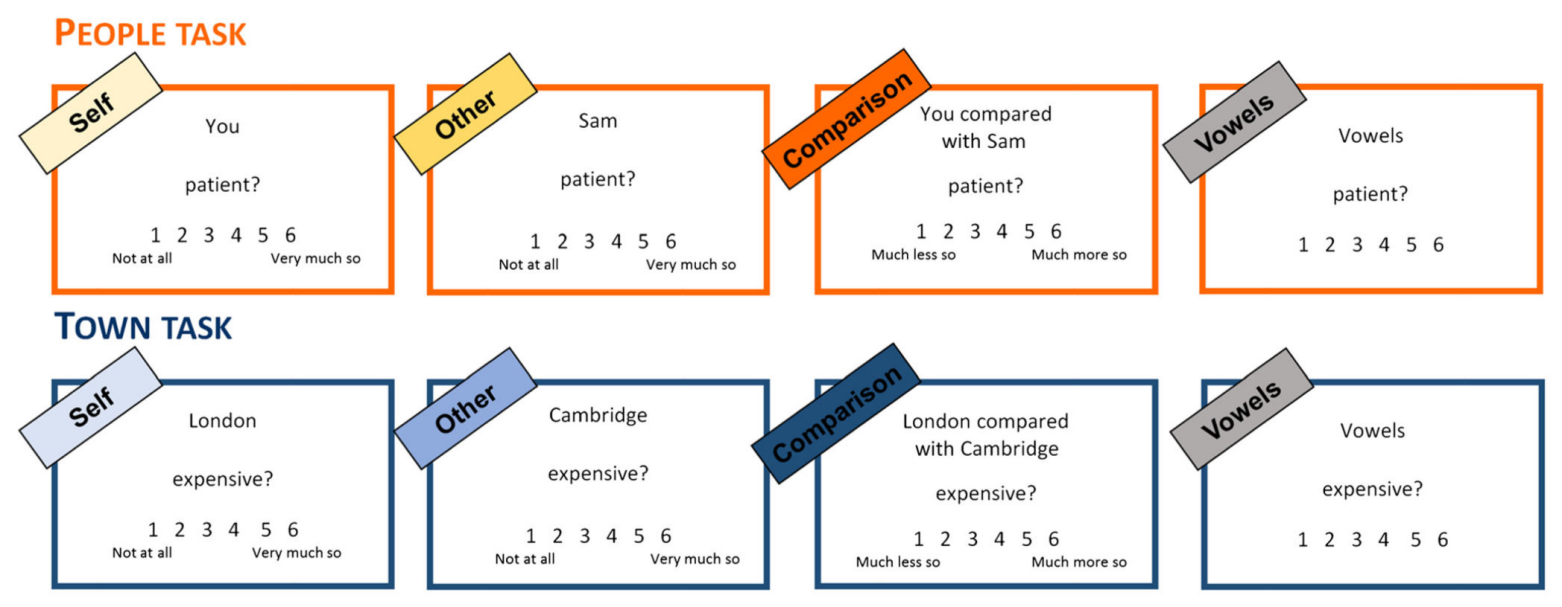
Example of stimuli for each task and condition. The Vowels condition was only included in Study 2. In the People task, participants were asked: “How much do you think the following words apply to you/your friend?” (Self/Other) or “How much do you think the following words apply to you compared with your friend?” (Comparison). On the rating scale, 1 indicated “not at all” and 6 indicated “very much so” in the Self and Other conditions, whereas 1 indicated “much less so” and 6 indicated “much more so” in the Comparison condition. All text was presented in white on a black background.

Judgments were blocked according to Task and Condition, and the order of the blocks was counterbalanced within and between participants. Each block started with an instruction screen indicating to participants what type of judgment they should make during that block. On each trial, this information was repeated at the top (e.g., “You”), an adjective was presented in the middle of the screen, and a rating scale from 1 to 6 was provided at the bottom of the scale (Figure 1).

Once participants had pressed a key to indicate their response, the corresponding number on the scale (1–6) was highlighted in red for 200 msec, followed by a 200-msec blank screen, and then a new trial started. There were 10 trials per block and 12 blocks in total, with two blocks of each of the six conditions (People or Town × Self, Other, or Comparison), that is, 20 trials in total per condition. A list of 20 adjectives was used for this study (fabulous, weird, loud, charming, romantic, crazy, pleasant, lovely, wild, perfect, busy, unique, friendly, cool, unusual, boring, dull, rich, quiet, and popular). All adjectives were presented once in each condition of each task.

### Data Analysis

Data were analyzed with SPSS 21 (IBM Corp., Armonk, NY). Median RTs were calculated for each Task and Condition. A 2 (Task) × 3 (Condition) × 7 (Age group) × 2 (Sex) mixed-model repeated-measures ANOVA (rmANOVA) was performed on the median RT data. Because of the nature of the task, a measure for accuracy is not available (we do not know objectively how “friendly” a participant or their friend is). Instead, we ran Spearman correlations to test whether participants were consistent in their response, that is, whether there was a positive correlation between their ratings in the Comparison judgment and the difference in ratings between the Self and Other judgments, across the set of 20 adjectives. For example, if a participant rated herself as “6” and her friend as “3” for “friendly,” a rating of “5” in the Comparison judgment (“How much more friendly do you think you are compared with your friend?”) would be consistent with the individual judgments. Spearman *r* correlation values obtained for each participant in each task were transformed using Fisher’s *z* transformation (*z* = 0.5 × ln ((1 + *r*)/(1 ‒ *r*)), resulting in consistency scores. A 2 (Task) × 7 (Age group) × 2 (Sex) mixed-model rmANOVA was performed on the consistency data. Post hoc comparisons investigating pairwise differences between age groups were Bonferroni corrected. Greenhouse–Geisser correction for nonsphericity was applied when necessary. Estimated means and standard errors from the rmANOVAs are reported in the text and in the figures.

### Results

#### RTs

A 2 (Task) × 3 (Condition) × 7 (Age group) × 2 (Sex) mixed rmANOVA showed a main effect of Condition (*F*(1.4, 435.6) = 500.00, *p* < .001, $\eta_{\text{p}}^{2}$ = 0.62), whereby participants responded faster in the Self (*M* = 1944 msec, *SD* = 36 msec) than the Other (*M* = 1989 msec, *SD* = 35 msec) condition (*p* = .042) and in both Self and Other conditions than in the Comparison condition (*M* = 2811 msec, *SD* = 56 msec; *p*s < .001); a main effect of Task (*F*(1, 311) = 12.04, *p* < .001, $\eta_{\text{p}}^{2}$ = 0.04), with slower RTs in the People (*M* = 2292 msec, *SD* = 44 msec) than the Town (*M* = 2203 msec, *SD* = 39 msec) task; and a main effect of Age group (*F*(6, 311) = 2.48, *p* = .024, $\eta_{\text{p}}^{2}$ = 0.05) but no main effect of Sex (*F*(1, 311) = 0.05, *p* = .818). Pairwise post hoc comparisons indicated that the 11- to 12-year age group responded more slowly than the 20- to 22-year age group (*p* = .019) and marginally slower than the 26- to 28-year age group (*p* = .081). No other pairwise comparison was significant.

**Figure 2:**
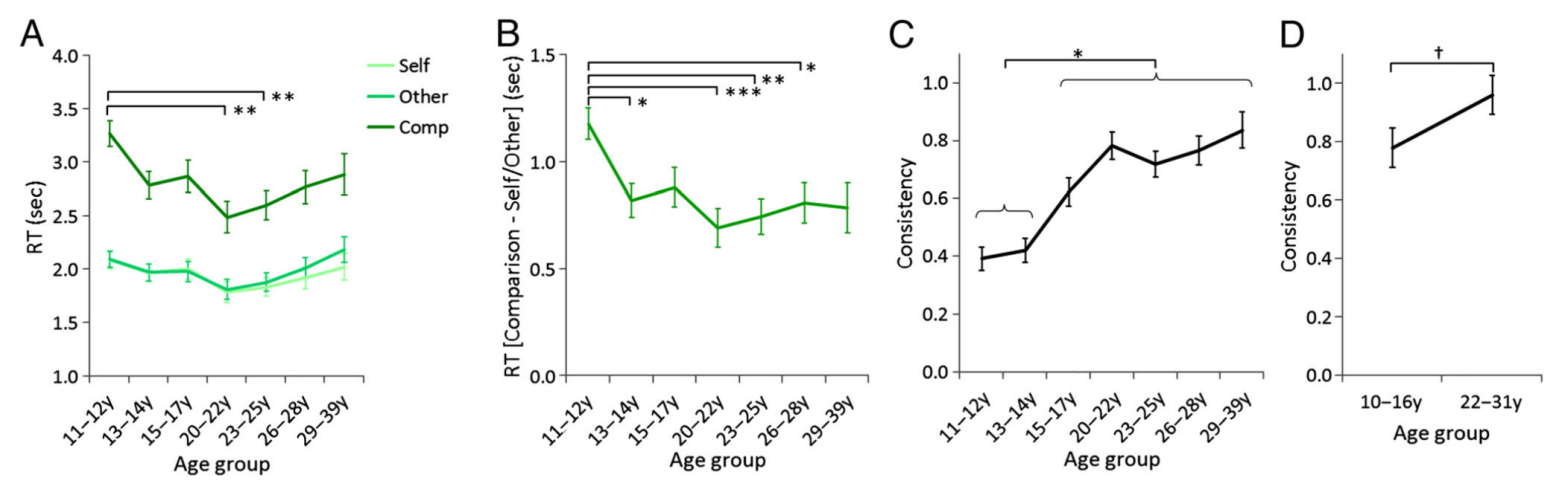
Mean RT and consistency scores as a function of age group. (A) Study 1: mean RTs as a function of Age group and Condition. (B) Study 1: mean difference in RTs between the Comparison and Self and Other conditions as a function of Age group. (C) Study 1: mean consistency scores as a function of Age group. (D) Study 2: mean consistency scores as a function of Age group. Error bars represent *SE*. ^†^*p* < .1, *p < .05, **p < .01, ***p < .001 (Bonferroni corrected). y = years.

The only significant interaction was between Condition and Age group (*F*(8.4, 435.6) = 3.64, *p* < .001, $\eta_{\text{p}}^{2}$ = 0.07). This was followed up by first entering the two 1-REL judgments in a 2 (Task) × 7 (Age group) × 2 (Sex) rmANOVA. This analysis showed no main effect of Age group (*F*(6, 311) = 1.54, *p* = .165) and no Age group × Condition interaction (*F*(6, 311) = 1.35, *p* = .233), indicating that the interaction was driven by the 2-REL, Comparison condition. Investigated separately, this condition showed a main effect of Age group (*F*(6, 311) = 3.61, *p* = .002, $\eta_{\text{p}}^{2}$ = 0.07). Post hoc comparisons indicated that the 11- to 12-year age group responded slower on average than the 20- to 22- and 23- to 25-year age groups (*p*s < .01; Figure 2A). To further investigate the interaction, the difference in median RT between the Comparison condition (2-REL, relational integration) and Self and Other conditions (1-REL judgments) was calculated. A 2 (Task) × 7 (Age group) × 2 (Sex) rmANOVA with post hoc pairwise comparisons indicated that the 11- to 12-year age group was relatively slower in 2-REL than 1-REL trials than the 13- to 14-, 20- to 22-, 23- to 25-, and 26- to 28-year age groups (*p*s < .05; Figure 2B).

#### Consistency

A 2 (Task) × 7 (Age group) × 2 (Sex) mixed rmANOVA was performed on consistency, which is the Fisher *z*-transformed Spearman correlation between participants’ answers in the Comparison condition and the difference between their answers in the Self and Other conditions. The main effect of Task was significant (*F*(1, 311) = 21.74, *p* < .001, $\eta_{\text{p}}^{2}$ = 0.07), with greater consistency in the Town (*M* = 0.713, *SD* = 0.025) than in the People (*M* = 0.586, *SD* = 0.021) task. In both tasks, mean consistency was positive, indicating some degree of consistency in participants’ responses across conditions. The main effect of Age group was also significant (*F*(6, 311) = 15.15, *p* < .001, $\eta_{\text{p}}^{2}$ = 0.23). No other main effect and no interaction were significant. Post hoc comparisons indicated that the 11- to 12- and 13- to 14-year age groups did not differ from each other and were less consistent than all older age groups (*p*s < .05), which did not differ from each other (Figure 2C).

In summary, participants were slightly slower in the Other than in the Self condition and in the People than in the Town task. However, there was a considerable difference in RT between 2-REL and 1-REL judgments, which decreased between 11–12 and 13–14 years old. Consistency was also higher in the Town than in the People task and improved between 13–14 and 15–17 years old. There was no interaction between Task and Age group, suggesting no evidence of a developmental difference in relational integration between the social and nonsocial tasks.

## Study 2: Neuroimaging Study

### Methods

#### Participants

Thirty-nine female participants aged between 10 and 31 years (*n* = 20 adults, *n* = 19 children and adolescents) took part in the neuroimaging study (Table 1). Seven adolescents and two adults had taken part in Study 1, with an interval of between 4 and 10 months between the testing sessions. Only female participants were included to reduce variability in the sample due to sex differences in brain development (Herting, Maxwell, Irvine, & Nagel, 2012; Raznahan et al., 2011). Sex differences were not found in Study 1 or in a previous behavioral and neuroimaging study of visuospatial relational reasoning development (Wendelken et al., 2011). However, other behavioral studies have reported sex differences in mentalizing (e.g., Charman, Ruffman, & Clements, 2002) and in relational reasoning (Lynn & Irwing, 2004). Because we were unable to collect a sample large enough to investigate sex differences, we chose to maximize the homogeneity of our sample by only including female participants.

Participants were reimbursed £20 and their travel expenses for taking part in the study. The study was approved by the UCL research ethics committee. Participants were divided into two groups, adolescents and adults. Adolescents were combined into a single group because of the sample size, with a focus on investigating the development of the neural correlates of relational reasoning, whereas Study 1 had focused on developmental changes in performance. The two groups were matched on estimated IQ (*t*(36) = 1.02, *p* = .314), which was assessed using the vocabulary and matrices subtests of the WASI (Wechsler, 1999).

#### Design and Stimulus Material

The fMRI task had two within-participant factors (Task: People or Town; Condition: Self, Other, Comparison, or Vowels) and one between-participant factor (Age group: adults, adolescents), resulting in a 2 × 4 × 2 mixed design. Participants were first trained on the task outside the scanner. After going through the four types of question for each Task, participants performed one block of three trials for each Task and Condition. Participants then performed four scanning runs as well as a structural scan between the second and third task runs. After scanning, participants completed the Interpersonal Reactivity Index (IRI; Davis, 1980), which provides measures of four components of empathy (empathic concern [EC], fantasy, personal distress, and PT), and were assessed on the WASI. The IRI was included as it has been used in previous neuroimaging studies as a measure of individual differences in social cognition in everyday life (Meyer et al., 2012; Raposo et al., 2011). Meyer et al. (2012) found an association between memory load-dependent activity within mentalizing regions and scores on the PT scale. We therefore aimed to relate activity within mentalizing regions in Study 2 with this everyday life measure of PT.

The paradigm was similar to the task described in Study 1, adapted from Raposo et al. (2011), with three differences. First, the fMRI task included a Vowels condition in which participants were required to count the number of vowels in the adjective presented on the screen (“Control” condition used by Raposo et al., 2011). This condition matched visual and verbal processing and motor execution demands while minimizing relational reasoning demands and, as such, was used as a baseline in the fMRI analyses. Regions of the social brain often show high activation at rest or during fixation phases, as part of the default mode network. Using an active baseline allowed the identification of activation of these brain regions, which was important for the People task. In addition, the words presented in the People and Town tasks differed, and using the Vowels condition as a baseline allowed us to control for BOLD signal differences elicited by the presentation of these words. Finally, using a similar baseline to Raposo et al. (2011) facilitates comparison of the results of the two studies. Second, traits were different for the People and Town tasks in an attempt to make them more relevant to each category. Third, a fixed ISI of 100 msec was used.

Participants performed two scanning runs of the People and Town task, alternating in an ABAB or BABA order, counterbalanced across participants. Each run was composed of five blocks of each condition (Self, Other, Comparison, and Vowels). The order of the conditions was fixed within a run and counterbalanced across runs and participants. After every Vowels block, there was a fixation block. Task blocks were preceded by a 1-sec instruction that specified the condition of the next block (e.g., “You,” “Sam,” “You compared with Sam,” “Vowels,” “London,” “Cambridge,” “London compared with Cambridge”) and was composed of three trials each.

Participants had a maximum of 6.05 sec to input their response on each trial, during which time the stimulus remained on the screen. When participants responded, the number they pressed turned red, and the stimulus remained on the screen until 6.1 sec after the onset of the trial presentation. A blank screen was displayed during the ISI. Stimuli consisted of lists of 30 adjectives in each task, which were matched for number of letters, number of vowels, frequency, and familiarity (see Appendix). Each adjective was presented once in each of the four conditions. Half of the adjectives were presented in the first scanning run of a task, half in the second run.

#### fMRI Acquisition

Multislice T2-weighted echo-planar volumes with BOLD contrast (35 axial slices with a voxel resolution of 3 × 3 × 3 mm covering most of the cerebrum, repetition time = 2.975 sec, echo time = 50 msec, acquisition time = 2.925 sec) were obtained using a 1.5-T MRI scanner (Siemens TIM Avanto, Erlangen, Germany). Functional images were acquired in four scanning runs lasting approximately 8 min 40 sec each in which 174 volumes were obtained. The first four volumes of each run were discarded to allow for T1 equilibrium effects. A 3-D T1-weighted fast-field echo anatomical image lasting 5 min 30 sec was acquired after the first two functional runs for each participant.

#### Data Analysis

*Behavioral data.* A 2 (Task) × 4 (Condition) × 2 (Age group) mixed rmANOVA was performed on median RT data. A 2 (Task) × 2 (Age group) mixed rmANOVA was employed to analyze mean consistency, which was calculated in the same way as in Study 1.

*MRI data.* MRI data were preprocessed and analyzed using SPM8 (Wellcome Trust Centre for Neuroimaging, London, United Kingdom; www.fil.ion.ucl.ac.uk/spm/). Images were realigned to the first analyzed volume with a second-degree B-spline interpolation to correct for movement during the session. The bias-field-corrected structural image was coregistered to the mean, realigned functional image and segmented on the basis of Montreal Neurological Institute (MNI)-registered International Consortium for Brain Mapping tissue probability maps. Resulting spatial normalization parameters were applied to the realigned images to obtain normalized functional images with a voxel size of 3 × 3 × 3 mm, which were smoothed with an 8-mm FWHM Gaussian kernel.

Realignment estimates were used to calculated frame-wise displacement (FD) for each volume, which is a composite, scalar measure of head motion across the six realignment estimates (Siegel et al., 2014). Volumes with an FD > 0.9 mm were censored and excluded from general linear model estimation by including a regressor of no interest for each censored volume. Scanning sessions with more than 10% of volumes censored or a root mean square (RMS) movement over the whole session greater than 1.5 mm (one session for three participants, two sessions for one participant) were excluded from the analysis. Adolescent and adult participants significantly differed in the number of overall censored volumes (*M*_adolescents_ = 3.39, *SD* = 3.61; *M*_adults_ = 0.19, *SD* = 0.38; *p* < .001), mean RMS translational movement (*M*_adolescents_ = 0.32 mm, *SD* = 0.11 mm; *M*_adults_ = 0.24 mm, *SD* = 0.07 mm; *p* = .005), and mean FD (*M*_adolescents_ = 0.18 mm, *SD* = 0.08 mm; *M*_adults_ = 0.10 mm, *SD* = 0.02 mm; *p* < .001). There was no difference between groups in terms of mean RMS rotational movement (*M*_adolescents_ = 0.23 mm, *SD* = 0.12 mm; *M*_adults_ = 0.17 mm, *SD* = 0.08 mm; *p* = .088).

Scanning runs were treated as separate time series, and each series was modeled by a set of regressors in the general linear model. Runs of the People or Town Task were each modeled by six box-car regressors: four regressors corresponding to each Condition (Self, Other, Comparison, and Vowels), with a duration of 18.6 sec; Instructions, with a duration of 1 sec; and Fixation blocks, with a duration of 18.6 sec except for the last block, which had a duration of 39 sec. All regressors were convolved with a canonical hemodynamic response function and, together with the separate regressors representing each censored volume and the mean over scans, comprised the full model for each session. The data and model were high-pass filtered to a cutoff of 1/128 Hz.

The second-level whole-brain analysis focused on relational integration, that is, the main effect of Comparison (2-REL) versus Self and Other (1-REL) conditions, and on differences between social and nonsocial tasks. The 1-REL conditions, Self and Other, were thus combined within each task. Four first-level contrasts were calculated using the Vowels condition as a baseline within each task: People (Self, Other) ‒ People Vowels (People SO), People Comparison ‒ People Vowels (People Comp), Town (Self, Other) ‒ Town Vowels (Town SO), Town Comparison ‒ Town Vowels (Town Comp). These contrasts were then entered into a random effects analysis using a Participant × Age group (2) × Block type (4) flexible factorial design, modeling Participant as a main effect (to account for the repeated-measure nature of the data) and the Age group × Block type interaction.

Main effects of Condition (Comparison > SO) and Task (People > Town and Town > People) and the interaction between the two factors and with Age group were determined using the *t* statistic on a voxel-by-voxel basis. Statistical contrasts were used to create SPMs thresholded at *p* < .001 at the voxel level and at family-wise error (FWE) corrected *p* < .05 at the cluster level (corresponding to a minimum cluster size of 77 voxels determined with SPM8). Activations that survived whole-brain FWE correction at *p* < .05 at the voxel level are indicated. All coordinates are given in MNI space. Significant interactions were followed up by extracting the mean signal across all voxels of significant clusters with MarsBar (Brett, Anton, Valabregue, & Poline, 2002) and analyzing simple effects in SPSS using *t* tests (with Bonferroni correction for multiple comparisons).

We performed exploratory correlation analyses between consistency in ratings and individual differences in activation in the tasks. Relevant task contrasts were entered in a two-sample *t* test design modeling the two age groups separately, with the behavioral measure as a single covariate of interest. Correlations were run between the contrasts [Comparison > SO] and [Comparison/Self/Other > Vowels] and the mean consistency across tasks, between the contrast [People Comparison > SO] and consistency in the People task, and between the contrast [Town Comparison > SO] and consistency in the Town task. In the same manner, we explored correlations between individual differences in the [People > Town] and [People Comparison > SO] contrasts and the PT scale of the IRI (see Meyer et al., 2015, for a similar approach).

### Results

#### Behavioral Results

*RTs.* There was a main effect of Task (*F*(1, 37) = 13.51, *p* = .001, $\eta_{\text{p}}^{2}$ = 0.27). In contrast to Study 1, participants were slower in the Town (*M* = 2419 msec, *SE* = 69 msec) relative to the People (*M* = 2282 msec, *SE* = 63 msec) task. There was a main effect of Condition (*F*(1.8, 66.8) = 29.17, *p* < .001, $\eta_{\text{p}}^{2}$ = 0.44; Table 2). Pairwise comparisons with Bonferroni correction revealed that participants were slowest in the Comparison condition relative to all other conditions (*M*_Comparison_ = 2638 ± 79 msec, *M*_Self_ = 2180 ± 67 msec, *M*_Other_ = 2196 ± 62 msec, *M*_Vowels_ = 2388 ± 77 msec; all *p*s < .005). The Vowels condition was the next slowest (all *p*s < .05). Self and Other conditions did not differ significantly from one another (*p* > .05). In contrast to Study 1, there was no main effect of Age group (*F*(1, 37) = 2.21).

There was a significant interaction between Task and Condition (*F*(3, 111) = 3.14, *p* = .028, $\eta_{\text{p}}^{2}$ = 0.08). This was followed up by analyzing the data in the People and Town tasks separately. In both the People and Town tasks, participants were slower in Comparison than Self and Other trials (all *p*s < .001). In the People task, participants were slower in Vowels than Self and Other trials (all *p*s < .05). The two-way interaction is driven by a greater difference between Comparison and Vowels trials in the Town task (*M*_Comp-Vowels_ = 342 msec) than in the People task (*M*_Comp-Vowels_ = 157 msec, *p* = .008), whereas the difference between Comparison and Self and Other trials did not differ between tasks (*p*s > .05). In contrast to Study 1, there was no significant interaction between Condition and Age group (*F*(1.8, 66.8) = 0.58). In line with Study 1, there were no significant interactions between Task and Age group (*F*(1, 37) = 1.43) or among Condition, Task, and Age group (*F*(2.7, 98.8) = 1.76).

*Consistency.* As in Study 1, participants were consistent overall, with positive correlations between the 1-REL and 2-REL ratings. Similar to the pattern in Study 1, there was a trend effect of Age group (*F*(1, 37) = 3.46, *p* = .071, $\eta_{\text{p}}^{2}$ = 0.09; Figure 2D), with lower consistency in adolescents than adults. There was also a main effect of Task (*F*(1, 37) = 18.04, *p* < .001, $\eta_{\text{p}}^{2}$ = 0.33): Consistency was lower for People than for Town (*M*_People_ = 0.74 ± 0.05, *M*_Town_ = 0.96 ± 0.07). As in Study 1, the interaction between Task and Age group was not significant (*F*(1, 37) = 0.18).

*IRI.* Analyses of the subscales of the IRI revealed significantly higher PT and EC scores for adults (*M*_PT_ = 19.5, *SE* = 0.91; *M*_EC_ = 20.9, *SE* = 1.1) than for adolescents (*M*_PT_ = 14.1, *SE* = 0.94; *M*_EC_ = 16.8, *SE* = 1.1; all *p*s < .05). Pearson correlation coefficients were computed to assess the relationship between PT and performance in the task. There was no correlation between PT and Consistency in the People task (*r* = −.187, *p* = .255). There was also no correlation between PT and mean median RT for the Self (*r* = −.021, *p* = .897), Other (*r* = .006, *p* = .973), or Comparison (*r* = .068, *p* = .680) conditions in the People task.

#### fMRI Results

Whole-brain analyses contrasted the Comparison condition to the combined Self and Other (SO) conditions in the People and Town tasks. The Vowels condition served as an active baseline.

**Figure 3:**
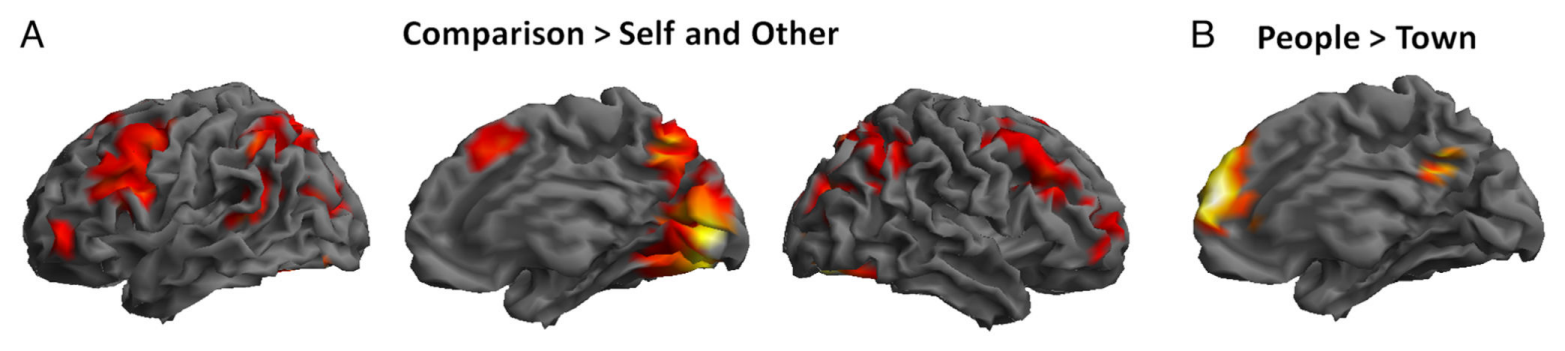
fMRI results across age groups. (A) Main effect of Condition. Regions showing increased BOLD signal in Comparison (2-REL) compared with Self and Other (1-REL) conditions are rendered on the SPM8 surface mesh template. From left to right: lateral view of the left hemisphere, medial and lateral views of the right hemisphere. (B) Main effect of Task. Regions showing increased BOLD signal in the People compared with the Town task are rendered on the SPM8 surface mesh template (medial view of the right hemisphere).

*Relational integration.* A broad bilateral network of frontoparietal, temporal, and occipital regions, including bilateral RLPFC, showed increased BOLD signal in Comparison versus SO conditions (Table 3 and Figure 3A), that is, in 2-REL as opposed to 1-REL processing. Increases in BOLD signal were observed in a large posterior cluster extending into bilateral occipital and lingual gyri, calcarine sulcus, and inferior parietal lobule and in anterior clusters in the precentral gyrus, pre-SMA, and inferior and middle frontal gyri.

#### Social Information Processing

When comparing the People task with the Town task, that is, when the information to be processed was of social (traits of participant or a friend) versus nonsocial (characteristics of towns) nature, increased BOLD signal was observed in clusters in the MPFC, insula, and precuneus (Table 3 and Figure 3B). The reverse contrast revealed increased BOLD signal in a large cluster extending into bilateral calcarine gyri, middle and superior occipital gyri, and bilateral clusters in the fusiform and parahippocampal gyri extending into the medial temporal gyri, middle cingulate cortex, bilateral precentral and postcentral gyri, and left inferior frontal cortex (Table 3).

**Figure 4:**
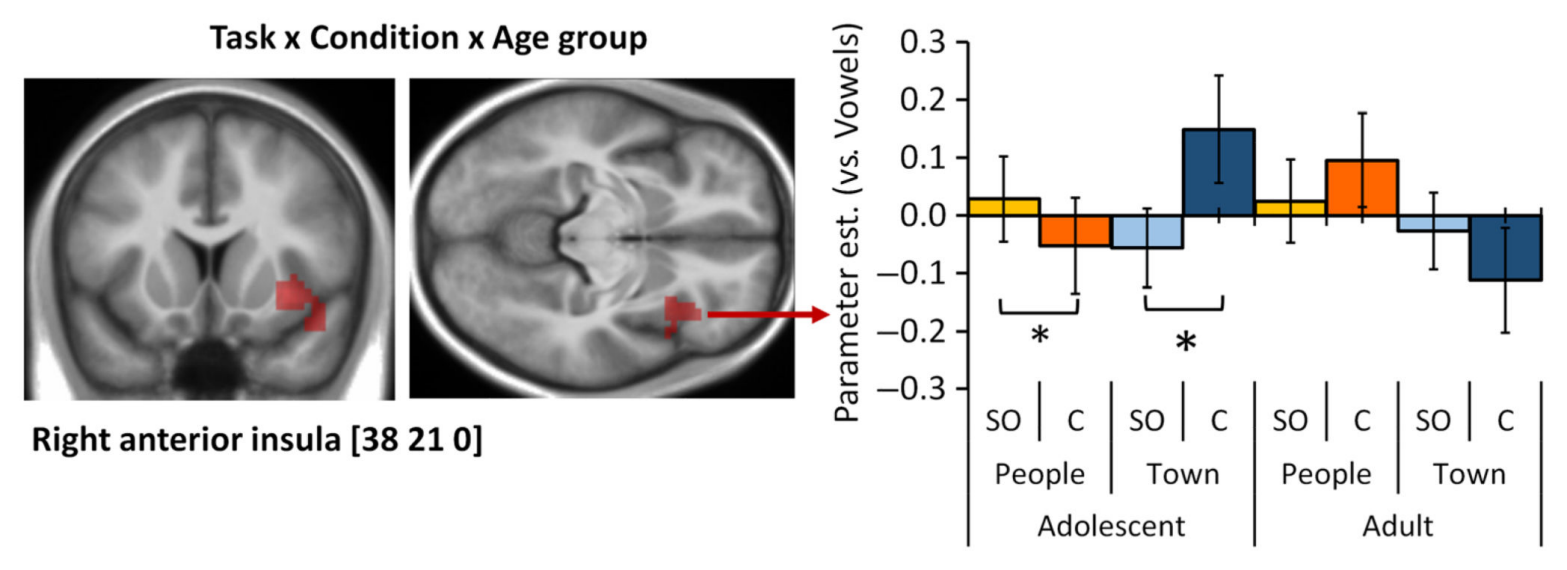
fMRI results of the interaction between Task, Condition, and Age group. On the left, the right anterior insula cluster showing a three-way interaction between Task, Condition, and Age group is shown on an average structural scan of all participants in the study (left: *y* = 21, right: *z* = 0). The contrast was thresholded at *p* < .001 uncorrected at the voxel level, *p*_FWE_ < .05 at the cluster level. On the right, parameter estimates extracted from this cluster are plotted in a bar chart to illustrate the three-way interaction. Error bars represent *SE*. ^†^*p* < .1, *p < .05, **p < .01, ***p < .001.

*Age group differences.* There was no two-way interaction between Task and Age group or between Condition and Age group; however, whole-brain analyses showed a significant three-way interaction between Task, Condition, and Age group in BOLD signal in the right insula (see Table 3 and Figure 4). The mean parameter estimates in this cluster were calculated, and the interaction was followed up by running 2 (Condition) × 2 (Age group) mixed rmANOVAs in each Task separately. There was an interaction between Condition and Age group in the People task (*F*(1, 37) = 7.20, *p* = .011, $\eta_{\text{p}}^{2}$ = 0.16) and in the Town task (*F*(1, 37) = 8.64, *p* = .006, $\eta_{\text{p}}^{2}$ = 0.19).

These interactions were further explored by comparing SO and Comparison conditions within each age group. In the People task, adolescents showed greater activation in the SO than the Comparison condition (*p* = .026), whereas the conditions did not differ in adults (*p* = .132). In the Town task, adolescents reversely showed greater activation in the Comparison than the SO condition (*p* = .018), whereas again, there was no difference in adults (*p* = .176).

#### Covariate Analyses

Whole-brain analyses were performed to investigate correlations between the behavioral measure of Consistency and BOLD signal during the task. No correlation between Consistency scores and individual differences in BOLD signal in the contrasts [Comparison > SO], [Comparison/Self/Other > Vowels], [People Comparison > People SO], and [Town Comparison > Town SO]) was observed. Further whole-brain analyses showed that BOLD signal in the [People > Town] and [People Comparison > People SO] contrasts was not significantly related to the PT scale of the IRI.

## Discussion

We performed separate behavioral and fMRI studies aiming to disentangle general and specific processes underlying relational integration of social information between late childhood and adulthood. We found behavioral evidence for general development of social and nonsocial relational reasoning. We discovered similar patterns of neural activity for adolescents and adults showing domain general involvement of the frontoparietal cortex areas associated with relational integration for both social and nonsocial relations and domain-specific involvement of the social brain for the manipulation of social information.

### Behavioral Findings

#### Relational Integration

In Study 1, the large behavioral study, we found earlier improvements in performance with age for RT and later improvements in consistency from late childhood to adulthood. Across age groups, RTs were slower in the Comparison condition, which required relational integration, than the Self and Other conditions, which required processing of a single relation. These results are in line with the robust processing speed costs observed in paradigms comparing relational integration with simpler relational processing (Dumontheil et al., 2010; Crone et al., 2009). In terms of relational integration performance, results show a pattern of early improvement in RT between ages of 11–12 and 13–14 years, with no further changes at older ages (Figure 2B), and improvements between ages of 13–14 and 15–17 years for the consistency measure, with no improvements at older ages (Figure 2C). Note that verbal IQ was not matched across age groups; however, the differences in verbal IQ did not directly map onto the observed age effects. In particular, there was no difference in IQ between 11- to 12-, 13- to 14-, and 15- to 17-year age groups, the age range where the key developmental changes were observed.

Similarly, in Study 2, the fMRI study, RTs were slower in the Comparison than in the Self and Other conditions. Relational integration was slower in adolescents than adults. There was also a trend for poorer consistency in the adolescents, which fits with the findings of the behavioral study. The less robust behavioral results in Study 2 compared with Study 1 were likely due to the smaller sample size in the imaging study.

Overall, we observed that late childhood and early adolescence are associated with poorer relational integration performance than adulthood (Figure 2B). This pattern fits with previous findings from visuospatial relational integration tasks, which indicate poorer relational integration accuracy in 8- to 12-year-olds than in adults (Crone et al., 2009), improvements in accuracy between 9- and 19-year-olds (Rosso, Young, Femia, & Yurgelun-Todd, 2004), and poorer combined accuracy and RT in 7- to 9-year-olds than in 14- to 17-year-olds (Dumontheil et al., 2010; reanalyzed in Dumontheil, 2014), although note that Wendelken et al. (2011) did not find age differences in 7- to 18-year-olds (see Dumontheil, 2014, for a review). This study is consistent with a previous investigation of the development of the integration of semantic information using an analogical reasoning task, which demonstrated poorer performance in 6- to 13-year-olds than in adults (Wright, Matlen, Baym, Ferrer, & Bunge, 2008).

### Social Information Processing

Developmental improvements in RT for relational integration did not vary as a function of the type of information. However, main effects of task across age were observed. In Study 1, RTs were faster overall for Town than People, whereas the opposite pattern was observed in Study 2 in which RTs were faster overall for the People task regardless of the level of relational processing, which is in line with previous findings of benefits in performance when stimuli are social rather than symbolic (Dumontheil, Hillebrandt, Apperly, & Blakemore, 2012; den Ouden, Frith, Frith, & Blakemore, 2005).

Both adolescents and adults showed increased speed for social information. Similarly, although consistency was overall greater in the nonsocial task, domain-general, not social-specific, developmental changes were observed. This is at odds with studies showing increased sensitivity to social stimuli during adolescence (Foulkes & Blakemore, 2016). This might be due to the low arousal and/or affective demands of the current task and should be explored in further studies. Furthermore, poorer overall consistency in the social task may be due to the greater complexity and variability of people’s traits compared with towns’ characteristics; this difference would apply to both adolescents and adults. For example, my friend Sam may be funny sometimes, but at other times, he is quite grumpy, while London is always busy.

Slight discrepancies between the findings from Studies 1 and 2 may be explained by methodological differences. Study 1 had a larger sample than Study 2, reducing the power needed to detect developmental changes in performance. In Study 1, the adjectives were the same for both the People and Town tasks. In Study 2, to maximize the mentalizing requirements of the People task, we used different adjectives for each task, which were more directly applicable to people or towns. The fMRI task was not self-paced. These factors may have affected the behavioral results.

Our behavioral findings provide some evidence for differential performance for social information, regardless of relational level, for both adolescents and adults. Furthermore, we provide evidence for domain-general development of relational integration of simple relations, which does not differ as a function of the social or nonsocial nature of the semantic information being processed.

### Neuroimaging Findings

#### Relational Integration

Relational integration was associated with greater activation in a large bilateral frontoparietal network including the RLPFC in both the People and Town tasks. A similar pattern of activation was observed by Raposo et al. (2011) in the Comparison versus Other contrast. These results further support the involvement of RLPFC and the inferior parietal cortex in relational integration (Wendelken et al., 2012; Crone et al., 2009). By adapting the task by Raposo et al., we are able to directly compare manipulation of social and nonsocial information and provide evidence for domain-general recruitment of the RLPFC through adolescence and adulthood across social and nonsocial domains.

Overall, we did not find evidence of an interaction between relational integration and social versus nonsocial task: Both networks were recruited in parallel for relational integration and social demands. This parallel recruitment of the two networks is similar to that observed by Meyer et al. (2012, 2015) in their social working memory task. However, these studies showed that MPFC activation associated with social working memory was modulated by working memory load (Meyer et al., 2012, 2015), whereas in this study, MPFC activation was not modulated by the number of relations participants had to consider. Similarly, although Meyer et al. (2012) found that PT on the IRI scale was positively associated with social working memory load-dependent activity within the MPFC and posterior cingulate cortex in adults, we found no association between self-reported PT on this questionnaire and behavior or brain activity in our task. A possible source of this difference in findings is that social cognitive load in the Meyer et al. (2012) study was higher than in this study, with the requirement to compare two, three, or four individuals, whose names were maintained in working memory, on given personality traits. In this study, our participants only compared two individuals, and the relevant information remained on the screen over the duration of the trial. These differences may have contributed to the lack of observed association between performance, MPFC activation, and self-reported real-life PT.

We did not observe developmental differences in activation in the RLPFC during relational integration across tasks in whole-brain analyses. Developmental differences have been reported in this brain region (e.g., Dumontheil et al., 2010; Crone et al., 2009). However, in the study by Crone et al., the age effects were mainly due to differences in time course of activations evident in their event-related design, which our block design did not allow us to test. In our previous study, age group effects were observed in ROI versus whole-brain analyses only (Dumontheil et al., 2010). Relational reasoning studies have traditionally involved demanding visuospatial reasoning tasks, such as the Raven Progressive Matrices. It might be that semantic reasoning about traits of people and towns is not demanding enough to tax adolescents in the same way.

Our results suggest domain-general recruitment of the RLPFC through adolescence and adulthood, independent of whether the information being manipulated is social or nonsocial. These results are therefore in line with the finding that the integration of visuospatial or semantic relations elicits similar activation of the relational integration network (Wendelken et al., 2012).

#### Social vs. Nonsocial Information Processing

Social information was associated with greater activation in the precuneus and MPFC in both adolescents and adults. These results are in line with a large body of literature that documents the involvement of these regions when processing social information (e.g., see Van Overwalle, 2009, for a meta-analysis). Note that, although towns can be considered social to some extent, as one can imagine the population of individuals living there, characteristics of towns were considered to be less social than traits of people.

Similar to this study, the investigation of social relational reasoning in adults by Raposo and colleagues (2011) reported greater MPFC activation in Self, Other, and Relational (Comparison) conditions when contrasted to a Vowel judgment condition. However, in contrast to our findings, Raposo et al. (2011) observed activation across ventral and dorsal MPFC in the Other versus Self contrast and no activation in the Self > Other contrast. This difference between the two studies may be due to the precise question participants were asked to answer. In this study, participants simply rated how funny (or other adjectives) they were, or their friend was, whereas in the Raposo et al. (2011) study, participants always rated how pleasant or unpleasant they found a concept (e.g., tower) or how pleasant or unpleasant they thought their friend would find this concept. It is possible that this latter question elicited greater mentalizing by asking participants to put themselves in their friend’s shoes rather than asking their own opinion. Overall, our results suggest social-specific recruitment of the MPFC during adolescence and adulthood for social information for both simple relations and integration across levels.

#### Interaction between Task, Condition, and Age Group

One neural difference between age groups was observed. At the cluster-corrected level, the right anterior insula showed a significant three-way interaction between Condition, Task, and Age group, driven by differential recruitment according to the domain (social vs. nonsocial) and the relational integration requirements with age. Although these results were not significant with an FWE-corrected threshold at the voxel level, they replicate the pattern of decreased activation with age in the anterior insula observed in a visuospatial relational reasoning task (Dumontheil et al., 2010). Functional changes in the anterior insula might reflect the maturation of neurocognitive strategies, which possibly include changes in task-specific connectivity between brain regions (e.g., see Bazargani et al., 2014; Dumontheil, 2014; and Dumontheil et al., 2010, for discussions). This neuroimaging finding does not directly map onto behavioral differences between the age groups. In a previous study, we similarly observed that developmental changes in anterior insula activation during relational reasoning were not accounted for by individual differences in performance on the task (Dumontheil et al., 2010). Beyond differences in the sensitivity of behavioral and brain imaging measures (e.g., evidenced by greater sensitivity of neuroimaging data than behavioral data to genetic differences; Dumontheil et al., 2011), differences may be due to the fact that behavior reflects a large combination of factors beyond the block-related activations measured in the current fMRI paradigm, such as event-related activations, which may have had compensatory effects on performance.

### Conclusion

We aimed to investigate the development of performance in social and nonsocial relational reasoning and their associated neural substrates. The paradigm required participants to make first- and second-order relational judgments about social and nonsocial information. Data from a behavioral study and an fMRI study demonstrated development of social and nonsocial relational reasoning in adolescence. These behavioral results with semantic stimuli are in line with previous research using mostly visuospatial relational reasoning tasks. We did not find evidence of differential development of relational integration of social versus nonsocial information in our behavioral studies. Similarly, the fMRI data showed that, in both adolescents and adults, relational integration of social and nonsocial information recruited a similar frontoparietal network. The processing of social information additionally engaged the MPFC and precuneus regions of the social brain, regardless of the order of reasoning. These findings provide further evidence that relational integration is a domain-general process (Wendelken et al., 2012).

## Figures and Tables

**Table 1:** Demographics of Participants in the Behavioral (Study 1) and fMRI (Study 2) Studies

		Sex	Age		Verbal IQ	
Age Group	N	F/M	Range	Mean (SD)	Range	Mean (SD)
*Behavioral study (Study 1)*						
11–12 years	66	39/27	11.10–12.98	12.05 (0.51)	87–137	114.4 (12.0)
13–14 years	57	30/27	13.06–14.97	13.92 (0.58)	84–140	113.1 (12.7)
15–17 years	42	20/27	15.01–17.99	16.18 (0.92)	84–134	114.0 (12.4)
20–22 years	43	20/22	20.34–22.97	21.93 (0.69)	97–137	118.1 (9.5)
23–25 years	50	28/22	23.01–25.96	24.55 (0.92)	81–137	113.7 (13.7)^a^
26–28 years	39	20/19	26.06–28.68	27.30 (0.81)	84–129	107.6 (12.2)^b^
29–39 years	28	10/18	29.00–39.39	33.22 (2.80)	94–129	115.5 (9.5)
*fMRI study (Study 2)*						
10–16 years	19	Female only	10.98–16.83	14.10 (1.89)	93–134	116.8 (11.4)
22–31 years	20	Female only	22.22–31.67	25.89 (2.76)	107–131	119.9 (6.7)^c^

a WASI data were missing for one participant.

b WASI data were missing for three participants.

c WASI data were missing for one participant.

**Table 2:** Mean RT and *SE* (msec) for Each Task (People, Town) and Condition (Self, Other, Comparison, Vowels) in Study 2

	People		Town	
	Adolescents	Adults	Adolescents	Adults
Self	2215 (110)	2057 (107)	2333 (100)	2115 (98)
Other	2123 (94)	2112 (92)	2378 (96)	2171 (94)
Comparison	2570 (108)	2460 (106)	2904 (136)	2618 (133)
Vowels	2503 (112)	2212 (109)	2529 (120)	2309 (117)

**Table 3:** Summary of Neuroimaging Results

	L/R	BA	MNI (x, y, z)	Z Score	Cluster Size
*Main effect of condition (Comparison > SO)*					
Lingual gyrus	L	18	−9, −85, −14	>8^a^	4,959^b^
Calcarine gyrus		17	0, −85, 1	>8^a^	
Lingual gyrus	L	18	−21, −79, −14	>8^a^	
Lingual gyrus	R	18	18, −79, −14	>8^a^	
Precuneus	R	7	3, −61, 46	>8^a^	
Middle occipital gyrus	R	19	30, −79, 19	7.31^a^	
Middle occipital gyrus	L	19	−30, −76, 22	6.53^a^	
Middle temporal gyrus	L	21	−48, −46, 10	6.22^a^	
Inferior parietal gyrus	L	40	−45, −46, 43	6.13^a^	
Fusiform gyrus	R	19	27, −64, −5	5.90^a^	
Inferior frontal gyrus	L	48	−48, 14, 25	6.59^a^	1,351^b^
Precentral and middle frontal gyri	L	6	−39, −1, 55	5.78^a^	
Middle cingulate cortex and pre-SMA	R	32	9, 20, 46	5.77^a^	
Inferior frontal gyrus	L	48	−36, 20, 22	5.68^a^	
Middle frontal gyrus	R	44	30, 14, 43	5.55^a^	819^b^
Inferior frontal gyrus	R	44	48, 26, 31	5.32^a^	
Middle frontal gyrus	R	8	30, 23, 52	5.03^a^	
Middle and superior frontal gyri	R	10	30, 59, 7	4.56^a^	
Precentral gyrus	R	6	30, −4, 46	4.44	
Inferior and middle frontal gyri	L	47	−39, 47, −2	4.91^a^	95^b^
*Main effect of task (People > Town)*					
MPFC	R	10	6, 53, 13	7.22^a^	1,649^b^
ACC	L	32	−3, 53, 13	7.11^a^	
ACC	L	10	−6, 44, 1	5.95^a^	
Anterior insula	R	48	30, 17, −17	5.74^a^	69
Precuneus and posterior cingulate cortex	R	23	6, −52, 28	5.26^a^	132^b^
*Main effect of task (Town > People)*					
Lingual gyrus	R	30	9, −49, 4	>8^a^	2,164^b^
Calcarine gyrus	R	30	15, −52, 13	7.65^a^	
Calcarine gyrus	L	30	−12, −55, 10	7.17^a^	
Middle occipital gyrus	L	19	−33, −76, 28	6.08^a^	
Middle occipital gyrus	R	19	36, −70, 37	5.90^a^	
Superior occipital gyrus	L	23	−21, −64, 28	5.25^a^	
Superior occipital gyrus	R	7	24, −76, 46	4.62^a^	
Fusiform and parahippocampal gyri	L	37	−30, −37, −14	>8^a^	192^b^
Fusiform gyrus	R	37	30, −31, −17	6.97^a^	302^b^
Inferior temporal gyrus	R	20	54, −46, −11	4.13	
Parahippocampal gyrus	R	35	21, −13, −20	3.73	
Middle cingulate cortex	R	23	9, −34, 34	4.78^a^	60
Precentral gyrus and inferior frontal operculum	L	44	−42, 8, 28	4.58^a^	181^b^
Postcentral and precentral gyri	R	43	60, −10, 31	3.93	108^b^
*Interaction adolescents > adults [(Town Comparison > SO) > [People (Comparison > SO)]*					
Anterior insula	R	48	33, 17, −8	4.26	178^b^
Anterior insula	R	47	39, 26, 4	3.71	
Superior temporal pole	R	38	45, 14, −20	3.59

Coordinates and *Z* scores are listed for regions showing a significant difference in BOLD signal for the main effect of Condition [Comparison > SO], the main effect of Task [People > Town] or [Town > People], and the interaction between Condition, Task, and Age Group [(Adolescents > Adults [(Town Comparison > SO) > [People (Comparison > SO)]]. Region labeling was done using automatic anatomical labeling (Tzourio-Mazoyer et al., 2002). BA labeling of peak of activations was done using MRIcron. L/R = left/right hemisphere.

a Voxels where *p*_FWE_ < .05 at the voxel level.

b Clusters where *p*_FWE_ < .05 at the cluster level, with a cluster-defining threshold of *p* < .001 uncorrected at the voxel level.

## Appendix

### List of Stimuli for each Task in Study 2

In the People task, participants were asked: “How much do you think the following words apply to you/your friend?” or “How much do you think the following words apply to you compared with your friend?” In the Town task, participants were asked: “How much do you think the following words apply to your town/other town?” or “How much do you think the following words apply to your town compared with the other town?” Familiarity and frequency measures were included to ensure that all adjectives were commonly used English words and that their occurrence was comparable between Tasks.

**Table:**

	People	Town	
Adjectives	arrogant, jealous, timid, selfish, careless, witty, cheerful, thoughtful, stubborn, ambitious, confident, aggressive, clever, smart, bold, brave, generous,helpful, mature, wise, tough, funny, curious, honest, sensitive, friendly, fair, patient, bright, happy	run-down, shabby, boring, rainy, noisy, sleepy, vibrant, picturesque, polluted, quaint, historic, lively, dull, exciting, romantic, dirty, urban, expensive, dangerous, rural, cultural, safe, unusual, clean, quiet, traditional, famous, amazing, flat, ancient	
Number of letters	*M* = 6.7, *SD* = 1.8	*M* = 6.6, *SD* = 1.9	*t*(58) = 0.14, *p* = .89
Number of vowels	*M* = 2.5, *SD* = 1.1	*M* = 2.6, *SD* = 1.1	*t*(58) = 0.36, *p* = .73
Familiarity	*M* = 541.6, *SD* = 48.6	*M* = 561.6, *SD* = 53.6	*t*(40) = 1.2, *p* = .23
Brown frequency	*M* = 9.26, *SD* = 10.6	*M* = 7.70, *SD* = 11.0	*t*(41) = 0.47, *p* = .64
Kucera–Francis frequency	*M* = 32.0, *SD* = 26.9	*M* = 35.3, *SD* = 27.2	*t*(58) = 0.47, *p* = .64
